# Congenital Hallux Varus: A Rare Forefoot Deformity

**DOI:** 10.7759/cureus.17995

**Published:** 2021-09-15

**Authors:** Mohd Yazid Bajuri, Azwan Zhafri Bashir Ridha, Husna Mohd Apandi, Faris Aiman Sarifulnizam

**Affiliations:** 1 Orthopaedics and Traumatology, Universiti Kebangsaan Malaysia Medical Centre, Kuala Lumpur, MYS

**Keywords:** surgical treatment, medial deviation, congenital, great toe, congenital hallux varus

## Abstract

Congenital hallux varus is a rare forefoot deformity presenting with a deviation of the great toe medially. There are various techniques for the treatment of congenital hallux varus described in the literature. We present a case of a 16-year-old boy with congenital hallux varus who underwent corrective surgery, which involved soft tissue and bony procedure for better functional and clinical outcomes.

## Introduction

Hallux varus is defined as a medial deviation of the great toe at the metatarsophalangeal (MTP) joint, which is presented in three planes: supination, medial deviation, and clawing of the great toe. It can be congenital or acquired secondary to trauma, surgery, or neuromuscular disease. Patients will experience pain and difficulty with shoe wear if this condition is left untreated. If this condition is detected early, patients will be advised for conservative treatment, which includes splinting of the toes and proper footwear with a wide toe box or padding over the medial aspect of the foot to avoid callus formation [1]. However, if conservative measures fail, there are numerous surgical techniques that involve soft tissue or bony procedure available for congenital hallux varus.

## Case presentation

A 16-year-old boy presented to us with a left congenital hallux varus deformity. Previously, the patient defaulted follow-up since he was not experiencing any pain and was able to do his daily activities as usual. But in the past one year, he had difficulty in putting on an enclosed shoe, experiencing pain over the medial aspect of the left great toe upon wearing shoes, and difficulty in doing physical activities. Therefore, the patient came to seek our attention for further management. There was no history of trauma or surgery to the left foot.

**Figure 1:**
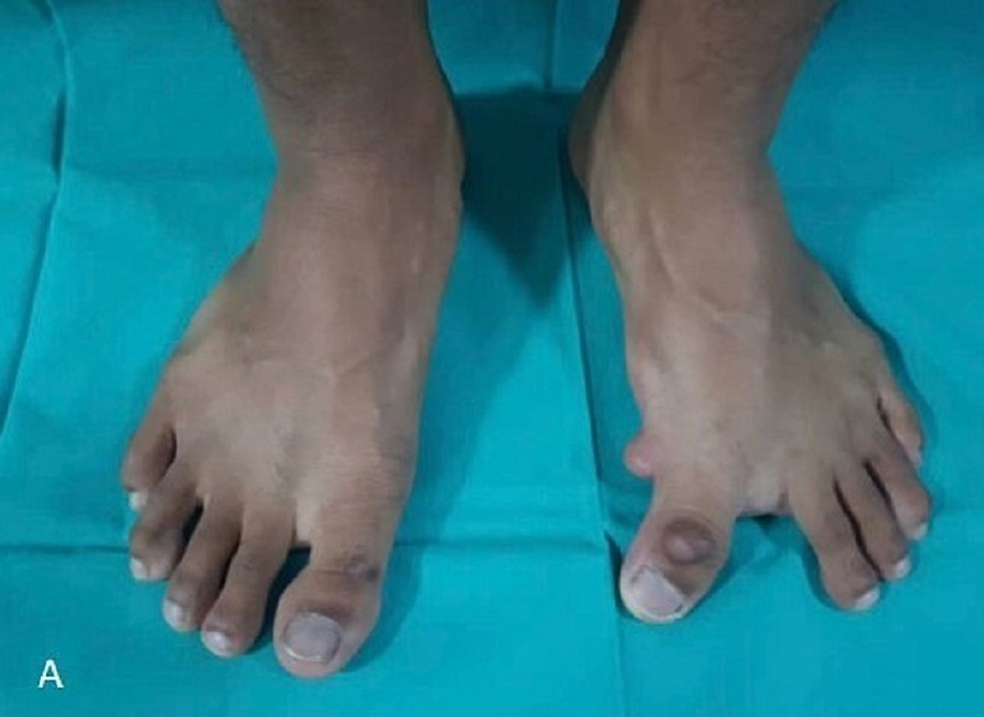
Preoperative clinical photo showing varus deformity of the left big toe with a polyp over the medial aspect of the left foot with significant enlargement of the first web space.

On examination, there was varus deformity of the left big toe with a polyp measuring 1 x 2 cm over the left foot at the medial aspect. The first web space was enlarged significantly (Figure 1). Dorsiflexion and plantarflexion of the first MTP joint were 30°.

**Figure 2:**
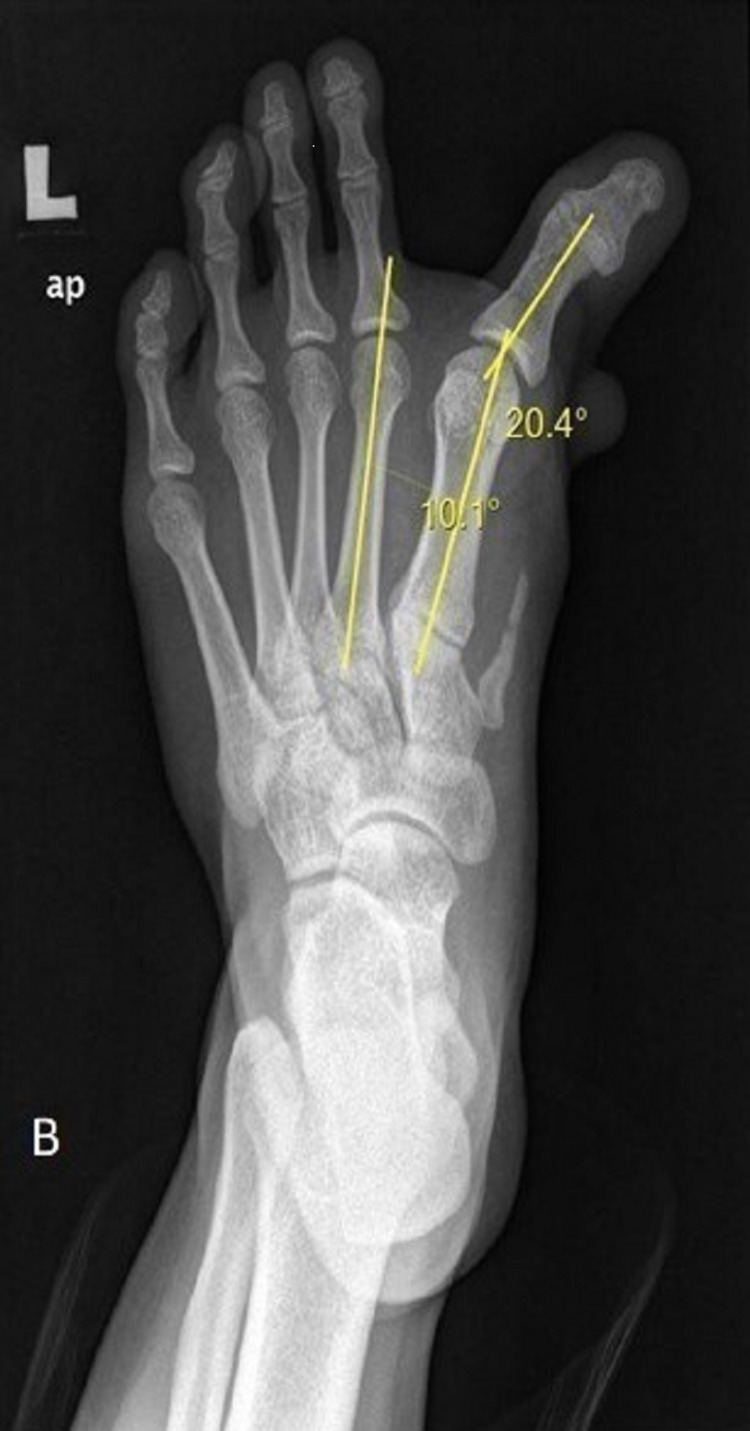
Preoperative X-ray of the left foot showing medial deviation of proximal phalanx of the great toe at the metatarsophalangeal (MTP) joint with an accessory bone over the medial cuneiform.

Radiographic imaging showed the medial deviation of the proximal phalanx of the great toe at the MTP joint with no bony deformity of the first metatarsal and phalanges of the great toe. There was an accessory bone over the medial cuneiform (Figure 2). Hallux varus angle was 20.4°; however, intermetatarsal angle (IMA) was normal (10.3°).

The patient underwent surgery and an incision was made over the medial aspect of the left foot overlying the proximal phalanx and first metatarsal bone. Skin polyp and accessory bone were removed. Tenotomy and lengthening of abductor hallucis, flexor hallucis longus, and extensor hallucis longus were performed. After that, we performed another small lateral incision at the proximal phalanx and proceed with a lateral closed osteotomy of the proximal phalanx and secured with a headless screw. The lateral capsule of the MTP joint was identified and plicated. Axial Kirschner wires (K-wires) of size 1.8 mm were inserted over the big toe and second toe in order to stabilize the soft tissue. For skin closure, a 3-0 Dafilon suture was used and sterile compression dressing was used for wound dressing. The foot was then protected with a boot slab.

**Figure 3:**
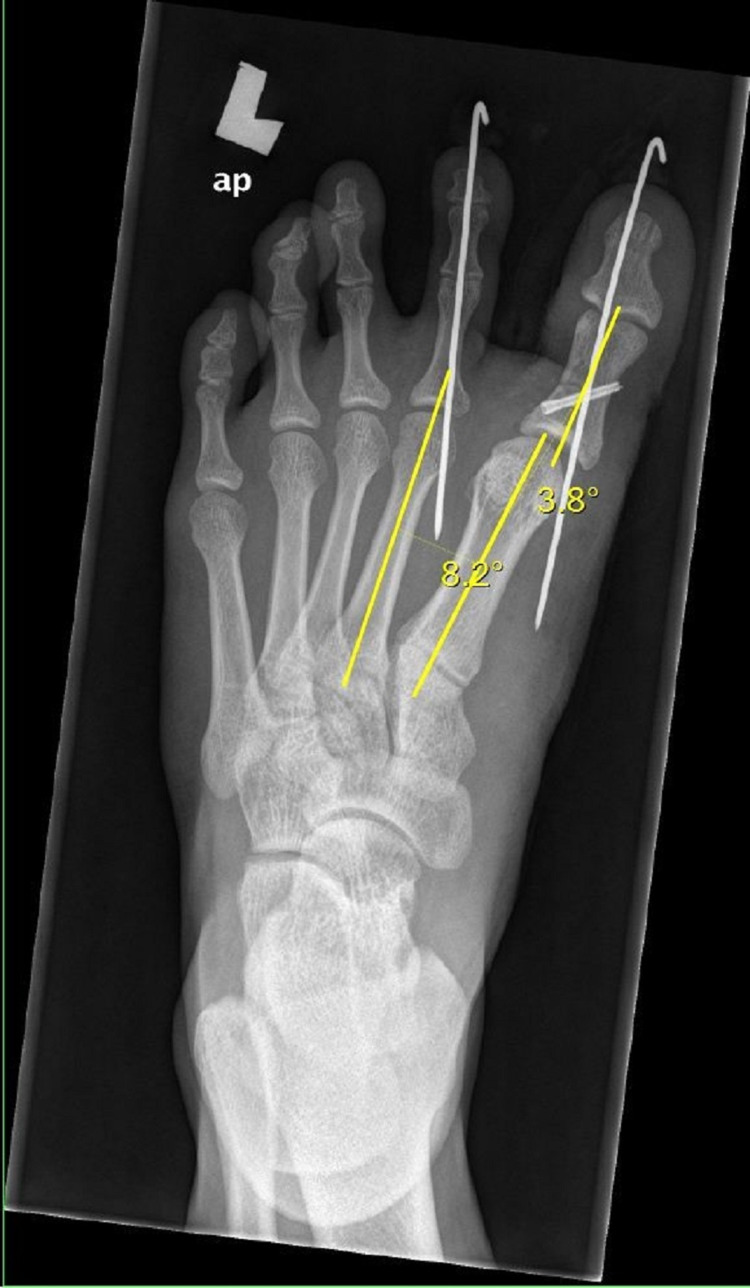
Immediate postoperative X-ray showing hallux varus angle and intermetatarsal angle (IMA) were reduced within the normal range.

**Figure 4:**
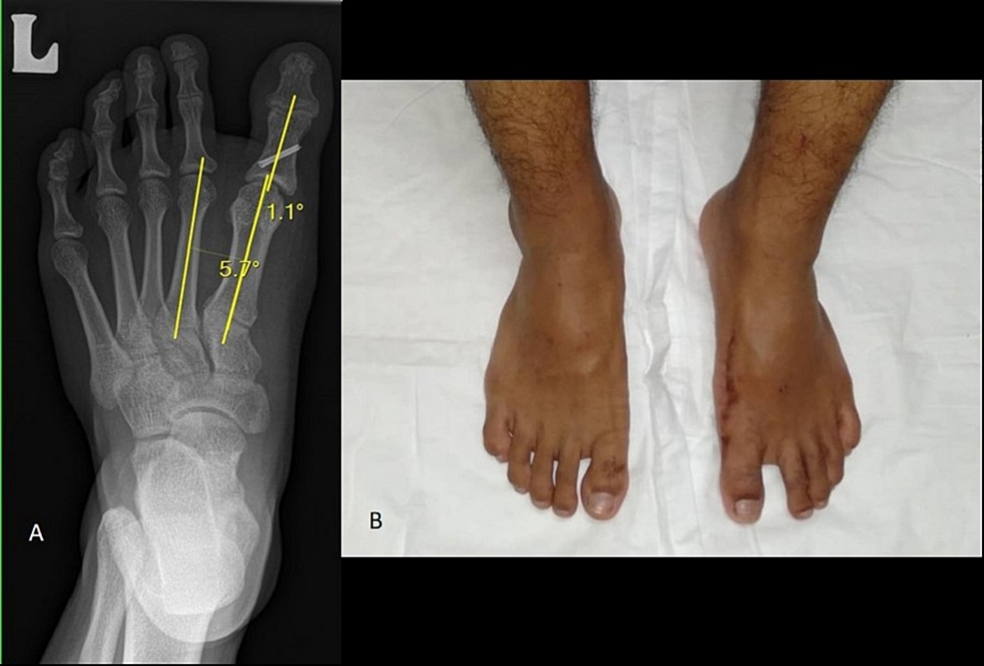
Four-month postoperative X-ray (A) and clinical photo (B) of the left foot showing evidence of union.

Postoperatively, the patient was well and imaging from the radiograph revealed the hallux varus angle and IMA were reduced within the normal range (Figure 3). Clinically, the wounds were clean and the patient was discharged five days after the surgery. After two weeks of surgery, the sutures were removed. Postoperatively, the patient was advised for non-weight-bearing for six weeks and a radiograph of his left foot was then reviewed, showing minimal callus formation. Therefore, he was continued for non-weight-bearing for another two weeks. In the eighth week, the K-wires were also removed and the patient was able to use regular shoes. The patient was then allowed for partial weight-bearing for about one month. A radiograph of his left foot was reviewed after three months, which showed that the osteotomy site was fully united and therefore, the patient was allowed for full weight-bearing. Four months after the surgery, he was allowed to run when clinical and radiological evidence of union was seen (Figure 4).

## Discussion

Congenital hallux varus is uncommon [1], whereby the great toe is medially angled at the metatarsophalangeal joint from a few degrees to as much as 90°. This deformity is characterized by supination, medial deviation, and clawing of the big toe, and also enlarged first web space [1]. The etiology of congenital hallux varus includes medial slope to the first metatarsocuneiform joints, medial cord thickening, shortened block first metatarsals, first metatarsal longitudinal epiphyseal bracket (LEB; delta phalanx), extra metatarsals occupying within the first web spaces, and ineffective abductor hallucis and adductor halluces insertions [2].

Morrissy et al. described the criteria to be assessed during the correction of hallux varus deformity: metatarsal-phalangeal incongruity correction, correction of the soft tissue tethered on the medial side of the hallux and enlarged first web space, metatarsal or bracket epiphyseal deformity correction if present, and correction of polydactyly if also present [3].

There are numerous surgical techniques reported in the literature. These techniques can be divided into soft tissue procedure, bony procedure, or a combination of both soft tissues with the bony procedure. The most common soft tissue procedures described in the literature are by McElvenny and Farmer. McElvenny proposed the removal of accessory bones, medial sesamoidectomy, and capsulotomy, medial fibrous band release, and lateral capsule reinforcement followed by transfixing of the metatarsophalangeal joint with a K-wire [4]. Farmer described rotational flap and skin flap and syndactylization involving the first and second toe [5]. Besides these two well-established techniques, there are few other procedures reported such as the entire abductor hallucis muscle and tendon resection, abductor hallucis tendon tenotomy, osteotomy of the metatarsal, and arthrodesis [6].

First metatarsal or proximal phalanx osteotomy was more commonly performed and the result was promising [7]. An osteotomy with lateral closing or medial open wedge of the first metatarsal or proximal phalanx plays an important part in the surgery of the congenital hallux varus. It can reduce varus angulation and cause relief of pain. Correction with bone grafting after the osteotomy is performed in case of short first metatarsal [8].

Shim et al. demonstrated that a combination of osteotomy with soft tissue procedure would be a good choice of treatment in the case of congenital hallux varus. They found out that after the soft tissue procedure, the first web space widening could still exist, and therefore, an osteotomy can be performed in order to correct any residual deformity [8].

In our case, the patient had contracted abductor hallucis with tight flexor hallucis longus and extensor hallucis longus that lead to deviation of the big toe medially at the metatarsophalangeal joint. However, there was no bony abnormality but the first web space was enlarged. Therefore, we decided to perform a lateral close osteotomy of the proximal phalanx with soft tissue procedure according to McElvenny: lengthening of the tendons, medial capsulotomy, and metatarsophalangeal joint transfixion with K-wires.

## Conclusions

A combination of both soft tissue and bony procedures is suggested to be the best option of treatment for congenital hallux varus regardless of the presence of any bony deformity as seen in this case. Correction of the hallux varus angle and widening of first web space are very essential for good clinical and cosmetic outcomes.
